# The Impact of Different High-Intensity Interval Training Protocols on Body Composition and Physical Fitness in Healthy Young Adult Females

**DOI:** 10.1089/biores.2018.0032

**Published:** 2018-12-28

**Authors:** Elise C. Brown, Tamara Hew-Butler, Charles R.C. Marks, Scotty J. Butcher, Myung D. Choi

**Affiliations:** ^1^ School of Health Sciences, Oakland University, Rochester, Michigan.; ^2^ College of Medicine, University of Saskatchewan, Saskatoon, Saskatchewan, Canada.

**Keywords:** HIIT, bone mineral density, bone mineral content, DXA scan, muscular fitness

## Abstract

Although traditional high-intensity interval training (HIIT) has been effective in improving body composition and physical fitness, it is unclear how multimodal HIIT affects these variables. This study compared the differences between these two training programs on body composition and physical fitness in apparently healthy, nonobese young adult females. A total of 16 participants (mean age = 23 ± 5.08 years) completed a 12-week HIIT intervention with two treatment groups: rowing and multimodal. Immediately before and after the intervention, the following measures were assessed: body mass index (BMI), total body mass, waist circumference, waist-to-height ratio, total body fat %, visceral adipose tissue, lean mass, bone mineral outcomes, cardiovascular fitness, and muscular fitness. A general linear model with repeated measures was used to assess changes over time for the group as a whole, as well as between-group differences. For the group as a whole, there were significant decrease in total body fat % (*p* = 0.04) and significant increases in BMI (*p* = 0.015), total body mass (*p* = 0.003), lean mass (*p* < 0.001), bone mineral content (BMC) (*p* < 0.001), VO_2_max (*p* = 0.01), broad jump (*p* = 0.001), squat endurance (*p* = 0.006), press (*p* < 0.001), back squat (*p* < 0.001), and deadlift (*p* < 0.001) one repetition maximum (1RM). The multimodal group (*p* < 0.001) increased deadlift 1RM significantly more than the rowing group (*p* = 0.002). HIIT can be an effective means for improving cardiovascular and muscular fitness, increasing lean mass and BMC, and thereby improving cardiometabolic as well as musculoskeletal health in nonobese females. Using a multimodal approach may give the added benefit of superior muscular strength increases.

## Introduction

Current international adult physical activity (PA) guidelines for health recommend a weekly minimum of 150 min of moderate-intensity aerobic activity and 2 days of muscular strengthening activities.^1^ However, these recommendations are time-consuming, and lack of time has been cited as a primary barrier to PA.^2^ High-intensity interval training (HIIT) may address this barrier. HIIT is exercise distinguished by short, intermittent periods of near-maximal exertion with rest or low-intensity activity between bouts, and requires a less time compared with continuous moderate-intensity exercise.^3^

HIIT can improve body fat outcomes^4^ but has not been effective in increasing lean mass in overweight/obese participants,^5^ and few studies have investigated HIIT on bone outcomes.^6,7^ In addition, although HIIT can effectively increase cardiovascular fitness,^8^ the impact of HIIT on muscular fitness (strength, power, endurance) is less known. This lack of evidence may be due to most HIIT protocols using traditional aerobic-type exercises such as running and cycling^8,9^ rather than resistance training exercises, which are generally associated with muscular fitness.

While both aerobics and resistance training can reduce total body fat % (BF%), visceral adipose tissue (VAT), blood pressure, and improve blood lipids,^9,10^ resistance training has the added benefits of improving physical function through increased muscular fitness, bone mineral density (BMD), and lean mass.^10^ Therefore, including resistance training exercises in an HIIT protocol may elicit additional health benefits compared with traditional HIIT.

An evolving form of HIIT, multimodal HIIT (MM-HIIT), uses resistance training exercises incorporating barbells, dumbbells, bodyweight movements, and other conditioning modalities.^11^ Over 6 weeks, MM-HIIT has resulted in greater muscular fitness and similar aerobic fitness adaptations compared with traditional HIIT.^11^ However, it is unclear how MM-HIIT compares with traditional HIIT over a longer time regarding physical fitness and body composition adaptations.

Therefore, the purpose of this study is to compare the effects of MM-HIIT and traditional HIIT using rowing (R-HIIT) over 12 weeks on body composition, cardiovascular fitness, and muscular fitness in apparently healthy, nonobese young adult females. It is hypothesized that both groups will similarly decrease body fat outcomes and increase aerobic fitness, and the MM-HIIT group will increase lean mass, bone mineral outcomes, and muscular fitness more than the R-HIIT group.

## Materials and Methods

Eighteen women were recruited from the intervention site, Oakland University in Rochester, Michigan. The intervention took place in the Oakland University Recreation and Well-Being Center. All participants were affiliates of Oakland University. The study design was a 12-week parallel-group randomized trial, and participants were randomized into either the MM-HIIT or R-HIIT group using a computerized random number generator. Although 16 of the 18 participants completed the intervention, 6 R-HIIT and 8 MM-HIIT group participants completed all pre- and postintervention measurements. Table 1 shows participant characteristics at baseline between groups.

**Table 1.** Participant Descriptive Statistics Between Groups at Baseline

	MM-HIIT group (*N* = 9)	R-HIIT group (*N* = 7)	*p*-Value (*t*-tests)
Age, years	23.78 ± 6.40	22.00 ± 2.83	0.507
Anthropometrics			
Height, cm	167.31 ± 6.65	156.54 ± 4.87 (*N* = 6)^a^	0.003^b^
Weight, kg	64.60 ± 9.83	60.39 ± 5.77 (*N* = 6)^a^	0.333
BMI, kg/m^2^	23.02 ± 2.69	24.68 ± 2.38 (*N* = 6)^a^	0.220
WC, cm	85.06 ± 5.81	83.70 ± 6.25	0.660
WHtR	0.51 ± 0.04	0.54 ± 0.05 (*N* = 6)^a^	0.245
DXA			
Total body fat %	34.49 ± 6.20	36.17 ± 6.08 (*N* = 6)^a^	0.596
VAT, cm^3^	55.68 ± 13.95	45.10 ± 19.24 (*N* = 6)^a^	0.222
BMC, g	2084.42 ± 310.90	1855.04 ± 162.75 (*N* = 6)^a^	0.100
BMD	1.05 ± 0.08	1.02 ± 0.05 (*N* = 6)^a^	0.318
Bone mineral T-score, SD	−0.67 ± 1.01	−1.09 ± 0.64 (*N* = 6)^a^	0.357
Bone mineral Z-score, SD	−0.62 ± 1.02	−1.06 ± 0.69 (*N* = 6)^a^	0.352
Fat mass, g	22754.06 ± 6175.00	38396.84 ± 39345.96 (*N* = 6)^a^	0.255
Lean mass, g	40501.36 ± 5555.71	36564.90 ± 3480.97 (*N* = 6)^a^	0.124
VO_2_max, mL/kg/min	30.33 ± 5.59	29.31 ± 7.68 (*N* = 6)^a^	0.762
Back squat endurance, repetitions	7.11 ± 6.75	11.71 ± 8.62	0.250
Broad jump, cm	130.36 ± 22.61	121.84 ± 17.57	0.426
Muscular strength			
Back squat 1RM, kg	43.16 ± 16.22	42.43 6.72	0.913
Press 1RM, kg	21.72 ± 3.43	19.81 ± 2.53	0.238
Deadlift 1RM, kg	61.62 ± 11.13 (*N* = 8)^a^	63.73 ± 7.86	0.683

All values are presented as mean ± SD.

^a^ Denotes different group sizes from column heading.

^b^ Denotes significant difference between groups.

1RM, one repetition maximum; BMC, bone mineral content; BMD, bone mineral density; BMI, body mass index; DXA, dual-energy X-ray absorptiometry; MM-HIIT, multimodal high-intensity interval training; R-HIIT, rowing high-intensity interval training; SD, standard deviation; VAT, visceral adipose tissue; WC, waist circumference; WHtR, waist-to-height ratio; VO_2_max, maximal oxygen consumption.

Inclusion criteria were nonobese (body mass index [BMI] ≤30 kg/m^2^), recreationally active females, ages 18–40 years, who did not engage in a systematic endurance or weight training program but had been participating in PA or exercise between 1 and 3 h a week for at least a month. Participants were excluded if they had an exercise-limiting cardiovascular, respiratory, metabolic, or musculoskeletal illness/injury or were taking medication that would alter the exercise response. This study was approved through the Oakland University Institutional Review Board. All participants provided informed consent before study involvement.

All participants had measures of body composition as the primary outcome variables, and cardiovascular and muscular fitness as secondary outcome variables assessed immediately before and after the intervention. All measurements took place on campus in the Oakland University School of Health Sciences Prevention Research Center and the Recreation and Well-Being Center. Assessments were performed by researchers and trained student researchers. The principal investigator was present for all data collection procedures.

Height and weight were taken with participants' shoes removed in light clothing using a mechanical stadiometer and scale. BMI was calculated from height and weight (kg/m^2^). Waist circumference (WC) was measured using a tape measure (Gulick II; Country Technology, Inc., Gays Mills, WI) placed directly on the skin, and the measurement site was the superior lateral border of the right ilium intersecting with the midaxillary line. Waist-to-height ratio (WHtR) was calculated from WC and height.

Total BF%, VAT, bone mineral content (BMC), BMD, bone mineral T-score, bone mineral Z-score, fat mass, and lean mass were assessed using a whole-body dual-energy X-ray absorptiometry (Hologic™ Horizon A, Marlborough, MA) scan.

All participants received three familiarization sessions introducing them to the movements used in the one repetition maximum (1RM) testing. Participants were tested for muscular strength using a 1RM for back squat, overhead press, and deadlift. A full back squat required the inguinal fold to drop below the superior portion of the patella; the press required the barbell to begin on the anterior deltoids and finish with elbows fully extended overhead; and the deadlift began with the weight on the floor and finished with the participant in an erect standing position with knees and hips fully extended.^12^ The researcher visually assessed each of these criteria and provided feedback to the participants. After performing a general and specific warm-up, a 1RM was achieved by increasing the resistance on individual attempts until the participant was unable to complete an attempt using proper technique. The general warm-up included 5 min of rowing at a light to moderate intensity, and the specific warm-up consisted of 8–10 repetitions of the tested 1RM exercise at a light weight, 3–5 repetitions at a moderate weight, and 1–3 repetitions at a moderate/heavy weight. The 1RM tests (back squat, press, deadlift) were conducted on separate days with 48 h between each test.

The back squat was used to estimate muscular endurance and the intensity used was equal to 70% of the participants' baseline 1RM back squat. The participants performed as many repetitions as possible, and the maximum number of repetitions was recorded.

Muscular power was assessed using the standing broad jump. Each participant received three trials with 2 min of rest between each trial. The best of the three trials was used.

Cardiovascular fitness was estimated by maximal oxygen uptake (VO_2_max in mL/kg/min) and was measured using a standard maximal cycling test performed on a mechanically braked cycle ergometer (Monark 828 E, Vansbro, Sweden). Respiratory gas analysis was obtained using a metabolic cart and mixing chamber. The TrueOne R metabolic system (ParvoMedics, Inc., Sandy, UT) was calibrated before each graded exercise test: the paramagnetic oxygen and infrared carbon dioxide analyzers were calibrated with a gas content of 16.00% oxygen, 4.00% carbon dioxide, and the balance nitrogen (Airgas Speedy Gases, Lenexa, KS). In addition, a 3.00 L syringe (Hans Rudolph, Inc., Kansas City, MO) was used to calibrate the pneumotachometer (Hans Rudolph, Inc.). The cycle ergometer was calibrated before testing preintervention and postintervention at a zero setting and then with a 2 kg calibration weight supplied by the manufacturer, Monark. Before testing, resting gas exchange data were collected for 3 min to obtain resting values of VO_2,_ followed by a 3-min warm-up at 20 watts (W). Participants initiated the cycling test, including 3-min stages for the first two stages with increasing intensity each stage of 30 W. The stages then decreased in duration to 1 min each, with increasing intensity each stage of 20 W. The cadence was maintained at 50 revolutions per minute each stage, and the test was terminated at volitional fatigue, defined as the participant no longer able to maintain 50 revolutions per minute for 20 sec. Tests were considered valid if the respiratory exchange ratio reached a minimum of 1.10, heart rate (HR) max ≥90% of age-predicted maximum HR, and/or a plateau in oxygen consumption with an increase in workload. VO_2_max was calculated using the mean of the highest five values over a 30-sec period.

For muscular fitness assessments, all participants completed these during the morning hours between 6am and 10am. All other assessments were conducted at varying times according to the participants' schedules. Due to limited equipment, the intervention was divided into two separate sessions. These sessions ran from 6:30am to 7:30am and from 7:30am to 8:30am.

The intervention included three weekly sessions for 12 weeks and was led by trained student researchers with the principal investigator present for all sessions. Participants were encouraged to attend a minimum of 80% of the sessions. Each session included a general and specific warm-up, the HIIT session, and a cool-down involving stretching. To attenuate the effects the warm-up may have had on training adaptations and to lessen the practice effect regarding 1RM testing,^13^ both groups completed identical warm-ups. The general warm-up consisted of 7 min of a light-/moderate-intensity circuit (45-sec work, 15-sec transition), including air squats, push-ups, lunges, kettlebell swings, kettlebell deadlifts, waiters' bows, and rowing. For the specific warm-up, three sets at increasing intensities (light, moderate, moderate/heavy) were performed for each exercise used in the MM-HIIT sessions that day (10, 5, and 3 repetitions for the first exercise; 10, 5, and 5 repetitions for the second exercise; and 5, 5, and 5 repetitions for the third exercise). For the training session, both groups completed six sets of 60 sec of all workouts, followed by 3 min of rest. Each set was completed with as much effort as possible across the full 60 sec (HR ≥90% of HR estimated max [220 − age] using HR monitor).^8^

For the MM-HIIT group, three movements were used in each set.^11^ The first movement was a barbell movement (e.g., press, squat) for 4–6 repetitions; the second movement was an assistance exercise utilizing dumbbell or bodyweight movements (e.g., lunges, bent-over rows) for 8–10 repetitions; and the third movement was a fast, sprint-like movement (e.g., hurdle hops, ball slams) for the remainder of the 60 sec. The weight/repetitions used for each exercise was recorded and intensity/speed was increased at subsequent sessions once maximum prescribed repetitions were reached. Each session had the same exercises within each set, but every subsequent session was different from the previous one for movements utilized. Eight different workouts were used that repeated themselves in the same order for the full 12 weeks (Supplementary Table S1).

The R-HIIT group trained using a rowing ergometer each set. Each training session was the same across the 12 weeks. The number of meters rowed was recorded each set and participants were encouraged to increase the number of meters rowed each session.

To gauge intensity, participants wore HR monitors each session (Polar FT1, Bethpage, NY). Weekly HR and rate of perceived exertion (RPE) utilizing Borg's 10-point scale were recorded for all participants each set.^14^

All participants were instructed to continue dietary and PA behaviors throughout the intervention similar to behaviors before the intervention. For diet, participants were specifically instructed to maintain a similar calorie intake, macronutrient (proteins, carbohydrates, and fats) ratios, and not to start taking any type of supplements for performance, recovery, fat loss, or related effects. To monitor PA outside of the study, each Friday, participants completed the Godin Leisure Time Physical Activity Questionnaire.^15^ This questionnaire was also completed at baseline and postintervention.

Independent sample *t*-tests were used to determine if differences between groups existed at baseline across outcome variables, and also to determine if PA levels differed between groups at week 1, week 6, and week 12 of the intervention. A general linear model with repeated measures was used to determine changes in the whole group and differences between groups over time. A Bonferroni correction with simple effects procedure was used for main effects and pairwise comparisons to analyze mean differences. Effect sizes (ESs) were based on partial eta squared (η_p_^2^) and were calculated for all significant *F*-ratios. ESs of 0.01 (small), 0.09 (medium) and 0.25 (large) were used. All participants who completed the intervention were included in the analyses. Statistical analyses were computed using IBM SPSS Statistics for Windows, version 25.0 (Armonk, NY). *p*-Values were set *a priori* at <0.05. Sample size was calculated using G*Power 3.1.9.2. Based on changes in the primary outcome of VO_2_max using HIIT, a power of 0.80, an α = 0.05, as estimated correlation of 0.80 between repeated measures, and a medium ES of 0.09, the estimated total sample size was 12 participants.^16^

## Results

**FIG. 1.**
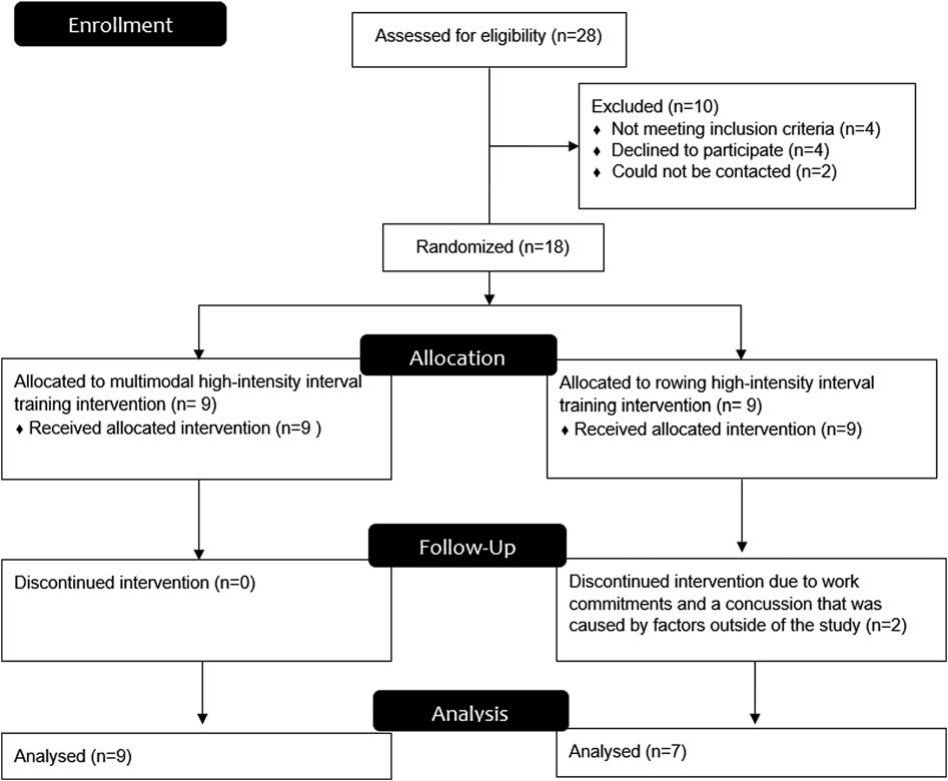
Flow of participants through the study.

Participants did not differ at baseline across any outcome variable (*p* > 0.05). Figure 1 shows flow of participants throughout the study. Sixteen participants completed pre- and postintervention assessments and were included in analyses, and compliance with the intervention was 84% for the MM-HIIT group and 81% for the R-HIIT group. PA outside of the study did not differ between groups at week 1 (*p* = 0.81), week 6 (*p* = 0.86), nor week 12 (*p* = 0.61). No adverse events were reported during testing, however, two muscle strains were reported throughout the intervention period (one from MM-HIIT and one from R-HIIT). These occurred outside of the exercise sessions and resulted in each participant missing one intervention session. HR was maintained at ≥90% of maximal HR throughout the intervention. The average RPE was 6.0, which corresponded between descriptors of “Hard (rating of 5)” and “Very Hard (rating of 7).”

The hypothesis that both groups would decrease adiposity outcomes similarly was not fully supported. From the general linear model with repeated measures, there was a significant increase for the whole group from pre- to postintervention for only BMI [*F*(1,13) = 7.76, *p* = 0.015, η_p_^2^ = 0.374] and total body mass [*F*(1,13) = 12.49, *p* = 0.003, η_p_^2^ = 0.471], and a decrease in total BF% [*F*(1,14) = 5.11, *p* = 0.04, η_p_^2^ = 0.267]. No significant interactions were present. There were no other significant changes in adiposity outcomes. See Table 2 for changes in body composition outcomes from pre- to postintervention for both groups.

**Table 2.** Body Composition Variables at Pre- and Postintervention for Multimodal High-Intensity Interval Training and Rowing High-Intensity Interval Training Groups

	MM-HIIT (*N* = 9)		R-HIIT (*N* = 6)			
Variable	Preintervention	Postintervention	Preintervention	Postintervention	Mean difference (95% CI)	*p*-Value (time × group)
Anthropometrics						
Weight, kg	64.6 ± 9.83	66.56 ± 10.8	60.39 ± 5.77	62.13 ± 6.26	0.08 (−2.253 to 2.413)	0.826
BMI, kg/m^2^	23.02 ± 2.69	23.63 ± 2.79	24.92 ± 2.51	25.35 ± 2.72	0.211 (−0.692 to 1.114)	0.641
WC, cm	85.06 ± 5.81	86.53 ± 7.66	83.70 ± 6.25 (*N* = 7)^a^	83. 47 ± 6.25 (*N* = 7)^a^	1.54 (−0.94 to 4.02)	0.163
WHtR	0.51 ± 0.04	0.52 ± 0.05	0.53 ± 0.06	0.53 ± 0.05	0.011 (−0.006 to 0.028)	0.403
DXA						
Total body fat %	34.49 ± 6.20	33.86 ± 6.73	36.17 ± 6.08	35.00 ± 6.46	0.615 (−1.165 to 2.395)	0.511
VAT, cm^3^	55.68 ± 13.95	53.72 ± 16.97	45.10 ± 19.24	47.83 ± 21.64	5.342 (−14.651 to 3.967)	0.257
DXA						
BMC, g	2084.42 ± 310.90	2124.67 ± 332.34	1855.04 ± 162.75	1882.13 ± 166.00	−5.233 (−35.365 to 24.899)	0.38
BMD, g/cm^2^	1.05 ± 0.08	1.06 ± 0.07	1.02 ± 0.05	1.02 ± 0.05	0.003 (−0.015 to 0.022)	0.927
BM T-score, SD	−0.67 ± 1.01	−0.64 ± 0.89	−1.09 ± 0.64	−1.10 ± 0.62	0.076 (−0.153 to 0.304)	0.764
BM Z-score, SD	−0.62 ± 1.02	−0.61 ± 0.89	−1.06 ± 0.69	1.04 ± 0.62	0.056 (−0.189 to 0.301)	0.981
Fat mass, g	22754.06 ± 6173.00	23763.9 ± 7041.65	22012.99 ± 4844.76	22222.86 ± 5693.70	575.395 (−768.40 to 1919.19)	0.286
Lean mass, g	40501.36 ± 5555.72	43254.38 ± 5490.18	36564.90 ± 3480.97	38734.63 ± 3419.02	790.891 (−257.672 to 1839.453)	0.232

Data are expressed as mean ± SD.

^a^ Denotes different group sizes from column heading.

BM, bone mineral; CI, confidence interval.

The hypothesis that the MM-HIIT group would increase lean mass and bone mineral outcomes more than the R-HIIT group was not supported. There was a significant increase for the whole group from pre- to postintervention in BMC [*F*(1,14) = 21.48, *p* < 0.001, η_p_^2^ = 0.605] and lean mass [*F*(1,14) = 111.23, *p* < 0.001, η_p_^2^ = 0.888]. However, no significant interactions were present.

For muscular fitness, the hypothesis that the MM-HIIT group would increase more than the R-HIIT group was partially supported. There was a significant increase for the whole group from pre- to postintervention on squat endurance [*F*(1,12) = 11.03, *p* = 0.006, η_p_^2^ = 0.479], broad jump [*F*(1,12) = 21.42, *p* = 0.001, η_p_^2^ = 0.641], back squat [*F*(1,12) = 40.45, *p* < 0.001, η_p_^2^ = 0.771], press [*F*(1,12) = 62.87, *p* < 0.001, η_p_^2^ = 0.840], and deadlift [*F*(1,12) = 60.28, *p* < 0.001, η_p_^2^ = 0.834]. There was also a significant interaction between groups for deadlift [*F*(1,12) = 4.85, *p* = 0.048, η_p_^2^ = 0.288] such that the MM-HIIT group increased [*F*(1,8) = 61.35, *p* < 0.001, η_p_^2^ = 0.89] more than the R-HIIT group [*F*(1,6) = 27.00, *p* = 0.002, η_p_^2^ = 0.82]. See Table 3 for changes in cardiovascular and muscular fitness outcomes from pre- to postintervention for both groups.

**Table 3.** Physical Fitness Variables at Pre- and Postintervention for Multimodal High-Intensity Interval Training and Rowing High-Intensity Interval Training

	MM-HIIT (*N* = 9)		R-HIIT (*N* = 7)		
Variable	Preintervention	Postintervention	Preintervention	Postintervention	Mean difference (95% CI)
Cardiovascular fitness					
VO_2_max, mL/kg/min	29.98 ± 5.87	31.63 ± 4.19	28.76 ± 8.27 (*N* = 6)^a^	32.09 ± 6.26 (*N* = 6)^a^	1.60 (−4.071 to 0.872)
Muscular endurance					
Squat endurance, repetitions	7.11 ± 6.75	15.00 ± 7.71	10.83 ± 9.09	20.50 ± 8.80	−4.21 (−13.39 to 4.978)
Muscular power					
Broad jump, cm	125.19 ± 17.59	142.18 ± 19.06	120.38 ± 18.78	135.02 ± 23.56	−0.253 (−14.324 to 13.818)
Muscular strength					
Back squat 1RM, kg	43.16 ± 16.22	52.15 ± 13.15	42.07 ± 7.29	49.50 ± 6.00	2.9 (−1.584 to 7.385)
Press 1RM, kg	21.72 ± 3.43	25.51 ± 3.73	19.32 ± 2.38	23.11 ± 2.66	0.476 (−1.821 to 2.774)
Deadlift 1RM, kg	61.62 ± 11.13 (*N* = 8)^a^	73.84 ± 12.82 (*N* = 8)^a^	63.52 ± 8.58	70.34 ± 8.09	5.831 (0.967 to 10.694)^b^

Data are expressed as mean ± SD.

^a^ Denotes different group sizes from column heading.

^b^ Denotes significance at the 0.05 level.

For cardiovascular fitness, the hypothesis that both groups would increase similarly was supported as there was a significant increase for the whole group from pre- to postintervention on VO_2_max [*F*(1,12) = 8.52, *p* = 0.01, η_p_^2^ = 0.415]. No significant interactions were found.

## Discussion

This was the first study, comparing MM-HIIT and traditional HIIT over a 12-week term, investigating changes in body composition and physical fitness. This intervention resulted in changes for the whole group, including increased total mass, lean mass, and BMC, with decreases in total BF%. Other changes included increased VO_2_max, squat endurance, broad jump, back squat, press, and deadlift 1RM. Also, deadlift 1RM increased more in the MM-HIIT group compared with the R-HIIT group.

Although no changes in WHtR, WC, fat mass, or VAT occurred, an increase in total mass and a decrease in total BF% for the whole group occurred. This likely resulted from the increased lean mass and BMC. Exercise-induced weight gain is metabolically favorable in healthy weight individuals, contrary to common beliefs that exercise-induced weight loss is necessary for improved health.^10^ Contrary to the present study, Trapp et al.^17^ found decreases in fat mass and trunk fat after a 15-week HIIT intervention in nonobese women. Some of the differences in findings may be due to divergent HIIT protocols between studies and the use of a diet inventory by Trapp et al.^17^ Heterogeneity has been reported across studies when examining different HIIT protocols on whole-body fat oxidation, which may help explain the discrepancies between studies.^18^ In addition, participants may have increased caloric intake in the present study. Participants were instructed to maintain similar dietary habits throughout the study as before the study, but exercise training has been found to increase appetite,^19^ which may have impacted caloric intake.

The unexpected similar increases in BMC for the MM-HIIT (+1.9%) and R-HIIT (+1.5%) groups suggest that both HIIT modalities with the specific warm-up provided a sufficient osteogenic stimulus. Increases in BMC are consistent with rowing and resistance training studies.^20,21^ Rowing combined with strength training and running occurring over 6–7 months has resulted in increased lumbar BMC^20^ and BMD,^21^ and was primarily attributed to the strain placed on lumbar spine during the drive phase of the rowing stroke. Increased bone mass is typically associated with high-impact and resistance training activities with high strain rates to produce an anabolic effect.^22^ Both HIIT protocols, including the specific warm-up, were a sufficient stimulus for increasing BMC in young adult females.

This study demonstrated unexpected similar lean mass increases of 1.96 kg (+6.8%) in MM-HIIT and 1.74 kg (+5.9%) in R-HIIT. Comparably, Blue et al.^23^ found increases in muscle cross-sectional area of the vastus lateralis after nine interval training sessions on a cycle ergometer in overweight/obese men and women. Although not directly measured, it can be speculated that both R-HIIT and MM-HIIT with the specific warm-up produced a strong enough stimulus to induce increases in myofibrillar protein synthesis alongside increased mitochondrial biogenesis.^24^ In addition, these lean mass increases may be clinically significant for many chronic diseases.^25^

Although increases across the whole group occurred for all muscular fitness variables, the MM-HIIT group (+19.8%) increased deadlift strength more than the R-HIIT group (+10.7%). These findings are inconsistent with the results of Buckley et al.^11^ who found no changes in muscular fitness for R-HIIT. The differences in findings between the two studies may be due to the warm-up protocol in the present study. While Buckley et al.^11^ used identical general warm-ups for both HIIT groups, the specific warm-ups were different. In the present study, both groups completed identical general and specific warm-ups, and this may have contributed to muscular fitness improvements. When considering the greater increase in deadlift 1RM in the MM-HIIT group, this finding may be due to a greater hormonal^26,27^ and neurological response from the deadlift compared with the back squat and press.^28^ While MM-HIIT is superior to traditional HIIT in increasing deadlift strength, traditional HIIT using rowing and a specific warm-up is also effective in improving muscular fitness.

It is well established that HIIT is an effective means of increasing aerobic capacity.^3,9^ Perhaps a more interesting finding was that MM-HIIT, using resistance exercises typically not associated with aerobic fitness, showed similar improvements in VO_2_max as traditional HIIT. This is consistent with a study by Buckley et al.^11^ that compared MM-HIIT and R-HIIT across 6 weeks in females.

This study has several limitations. The first limitation was the small sample size. Future studies should incorporate a multicenter strategy to increase the number of participants. Another limitation was the lack of a control group. Although efforts were made to include a control group, restricting recruitment efforts to only university affiliates limited the reach and resulted in too few participants to justify a control group. Including a control group in future studies would reduce the effects of confounders that may have influenced study outcomes. Also, the lack of familiarization to broad jump testing may have influenced improvements in this test due to a learning effect. Finally, the use of predicted maximum HR instead of measured maximal HR was used. Given that there was a wide variation in measured maximal HR, for simplicity during the intervention sessions, predicted maximal HR was used as most of the participants were of a similar age. While the results of this study are promising for populations, including those with obesity or cardiometabolic conditions, they should not be generalized to populations other than young, healthy, nonobese individuals. More research is needed with these other populations to determine the applicability of these protocols.

## Conclusion

HIIT has become a popular strategy to improve aerobic fitness, reduce adiposity, and improve cardiometabolic health in overweight and obese populations. This study highlights the important role that HIIT can also play in improving muscular fitness, increasing lean mass and BMC, and thereby improving cardiometabolic as well as musculoskeletal health in nonobese populations. Also, MM-HIIT has the added benefit of increasing muscular strength to a greater degree than R-HIIT. As the relationship between muscular fitness and the pathogenesis of cardiometabolic and certain musculoskeletal diseases becomes more appreciated, MM-HIIT interventions may play an important role in primary prevention throughout the life span.

## Supplementary Material

Supplemental data

## Abbreviations Used

1RM: one repetition maximum
BF%: body fat %
BM: bone mineral
BMC: bone mineral content
BMD: bone mineral density
BMI: body mass index
CI: confidence interval
DXA: dual-energy X-ray absorptiometry
ES: effect size
HIIT: high-intensity interval training
HR: heart rate
MM-HIIT: multimodal high-intensity interval training
PA: physical activity
R-HIIT: rowing high-intensity interval training
RPE: rate of perceived exertion
SD: standard deviation
VAT: visceral adipose tissue
WC: waist circumference
WHtR: waist-to-height ratio
