# Supplementary material for: The Impact of Different High-Intensity Interval Training Protocols on Body Composition and Physical Fitness in Healthy Young Adult Females
Source: Biores Open Access. 2018 Dec 28;7(1):177–85. doi: 10.1089/biores.2018.0032 (PMC6323591; doi:10.1089/biores.2018.0032)
Supplement: Supplemental data [file Supp_Table1.pdf]

Supplementary Data

Supplementary Table S1. Multimodal High-Intensity Interval Training Program

Monday	Wednesday	Friday
In 60 sec complete	In 60 sec complete	In 60 sec complete
Week 1		
4–6 Back squats<br>8–10 Pronated DB bent-over rows<br>Broad jumps for the remainder of the 60 sec	4–6 Bench press<br>8–10 DB stationary lunges<br>Ball slams for the remainder of the 60 sec	4–6 Deadlift<br>8–10 DB push press<br>Jump rope
Week 2		
4–6 Press<br>8–10 Supinated DB bent-over rows<br>Burpees for the remainder of the 60 sec	4–6 Front squats<br>8–10 Push-ups<br>Lateral hurdle hops for the remainder of the 60 sec	4–6 Incline bench press<br>Step-ups<br>KBS for the remainder of the 60 sec
Week 3		
4–6 Clean grip RDL<br>8–10 DB bench press<br>Ball slams for the remainder of the 60 sec	4–6 Push press<br>8–10 DB RDL<br>Broad jumps for the remainder of the 60 sec	4–6 Back squats<br>8–10 Pronated DB bent-over rows<br>Broad jumps for the remainder of the 60 sec
Week 4		
Bench press<br>8–10 DB stationary lunges<br>Ball slams for the remainder of the 60 sec	4–6 Deadlift<br>8–10 DB push press<br>Jump rope	4–6 Press<br>8–10 Supinated DB bent-over rows<br>Burpees for the remainder of the 60 sec
Week 5		
4–6 Front squats<br>8–10 Push-ups<br>Lateral hurdle hops for the remainder of the 60 sec	4–6 Incline bench press<br>Step-ups<br>KBS for the remainder of the 60 sec	4–6 Clean grip RDL<br>8–10 DB bench press<br>Ball slams for the remainder of the 60 sec
Week 6		
4–6 Push press<br>8–10 DB RDL<br>Broad jumps for the remainder of the 60 sec	4–6 Back squats<br>8–10 Pronated DB bent-over rows<br>Broad jumps for the remainder of the 60 sec	4–6 Bench press<br>8–10 DB stationary lunges<br>Ball slams for the remainder of the 60 sec
Week 7		
4–6 Deadlift<br>8–10 DB push press<br>Jump rope	4–6 Press<br>8–10 Supinated DB bent-over rows<br>Burpees for the remainder of the 60 sec	4–6 Front squats<br>8–10 push-ups<br>Lateral hurdle hops for the remainder of the 60 sec
Week 8		
4–6 Incline bench press<br>Step-ups<br>KBS for the remainder of the 60 sec	4–6 Clean grip RDL<br>8–10 DB bench press<br>Ball slams for the remainder of the 60 sec	4–6 Push press<br>8–10 DB RDL<br>Broad jumps for the remainder of the 60 sec
Week 9		
4–6 Back squats<br>8–10 Pronated DB bent-over rows<br>Broad jumps for the remainder of the 60 sec	4–6 Bench press<br>8–10 DB stationary lunges<br>Ball slams for the remainder of the 60 sec	4–6 Deadlift<br>8–10 DB push press<br>Jump rope
Week 10		
4–6 Press<br>8–10 Supinated DB bent-over rows<br>Burpees for the remainder of the 60 sec	4–6 Front squats<br>8–10 Push-ups<br>Lateral hurdle hops for the remainder of the 60 sec	4–6 Incline bench press<br>Step-ups<br>KBS for the remainder of the 60 sec
Week 11		
4–6 Clean grip RDL<br>8–10 DB bench press<br>Ball slams for the remainder of the 60 sec	4–6 Push press<br>8–10 DB RDL<br>Broad jumps for the remainder of the 60 sec	4–6 Back squats<br>8–10 Pronated DB bent-over rows<br>Broad jumps for the remainder of the 60 sec
Week 12		
4–6 Bench press<br>8–10 DB stationary lunges<br>Ball slams for the remainder of the 60 sec	4–6 Deadlift<br>8–10 DB push press<br>Jump rope	4–6 Press<br>8–10 Supinated DB bent-over rows<br>Burpees for the remainder of the 60 sec
